# p53 activates miR-192-5p to mediate vancomycin induced AKI

**DOI:** 10.1038/srep38868

**Published:** 2016-12-12

**Authors:** Jinwen Chen, Juan Wang, Huiling Li, Shixuan Wang, Xudong Xiang, Dongshan Zhang

**Affiliations:** 1 Department of Emergency Medicine, Second Xiangya Hospital, Central South University, Changsha, Hunan, People’s Republic of China; 2 Emergency Medicine and Difficult Diseases Institute, Central South University, Changsha, Hunan, People’s Republic of China; 3 Department of Ophthalmology, Second Xiangya Hospital, Central South University, Changsha, Hunan, People’s Republic of China; 4 Department of cellular Biology and anatomy, Medical college of Georgia at Georgia Regents University; Charlie Norwood VA Medical Center, Augusta, GA, USA

## Abstract

Pathogenic role of p53 in AKI remains controversial and the underlying mechanism is unclear. Here, we tested whether the inhibition of p53 may ameliorate vancomycin (VAN) induced acute kidney injury (AKI). Mice with p53 knock out (p53-KO) were resistant to VAN induced AKI, indicated by the analysis of renal function, histology, and apoptosis. Mechanistically, AKI was associated with the upregulation of several known p53 target genes, including Bax and p21, and this association was attenuated in p53-KO mice. Furthermore, the expression of miR-192-5p was significantly decreased in the p53-KO kidney tissues. In human renal tubular epithelial cell line (HK-2), VAN induced p53 accumulation and miR-192-5p expression. Both apoptosis of HK-2 cells and expression of miR-192-5p were also suppressed by pifithrin-α. Anti-miR-192-5p significantly blocked VAN-induced apoptosis and caspase activity in HK-2 cells. Consistently, *in vivo* inhibition of miR-192-5p also suppressed VAN induced AKI. Thus, we provided clinical and genetic evidence that p53 was associated with the development of VAN induced AKI through upregulation of miR-192-5p.

Vancomycin (VAN) is one of the most commonly used and most potent glycopeptide antibiotics^1^. It is being used for the treatment of severe Gram-positive infections caused mainly by Staphylococcus epidermidis, and methicillin-resistant Staphylococcus aureus (MRSA)^2^,^3^. The use of VAN is limited by its side effects in normal tissues, particularly nephrotoxicity^4^. Early impure VAN preparations (called ‘Mississippi mud’) induces higher nephrotoxicity, while purified VAN nephrotoxicity is rare^5^,^6^. However, VAN resistance with consequent treatment failure is progressively increased in staphylococci^7^. Therefore, one guideline suggested a dose of 15–20 mg/ml VAN. However, emerging data to achieve these treatment targets carry a substantial risk for nephrotoxicity^8^,^9^. Although some authors reported that the mechanism of VAN nephrotoxicity is similar to that of gentamicin, it remains unclear irrespective of numerous studies over the past several decades.

Recent studies demonstrated that apoptotic cell death plays a critical role in the pathogenesis of VAN induced acute kidney injury (AKI)^4^, which directly leads to renal cell damage and subsequent decline of renal function^2^,^10^. As we know that p53 is a tumor suppressor and can be induced by cancer and cellular stress in normal cells. Under various pathophysiological conditions, p53 may lead to cell cycle arrest and/or cell death, depending on the severity of DNA damage. However, the pathologic role of p53 in AKI remains controversial. We and others demonstrated that p53 plays a pathologic role in cisplatin-induced AKI using both cell culture and animal models including global p53 knockout mice^11^,^12^,^13^,^14^,^15^. p53 is also involved in kidney injury induced by aristolochic acid, folic acid, and glycerol injection^16^,^17^,^18^. However, as leukocyte p53 is renoprotective owing to the anti-inflammatory function, ischemic AKI is exacerbated by pifithrin-a and global p53 deletion in mice^19^. These date suggested that the role of p53 is associated with the cell type and AKI models.

In view of these findings, this study was initiated to assess whether inhibition of p53 can block VAN mediated AKI by using pharmacological and genetic inhibitory approaches. We demonstrate that blockade of p53 leads to the attenuation of VAN mediated AKI, further supporting a role of p53 in AKI. We further show that p53 may induce injury via miR-192-5p. Thus, targeting the p53-miR-192-5p might be a novel therapeutic strategy for VAN mediated AKI.

## Results

### VAN induced p53 accumulation in mice kidneys

**Figure 1:**
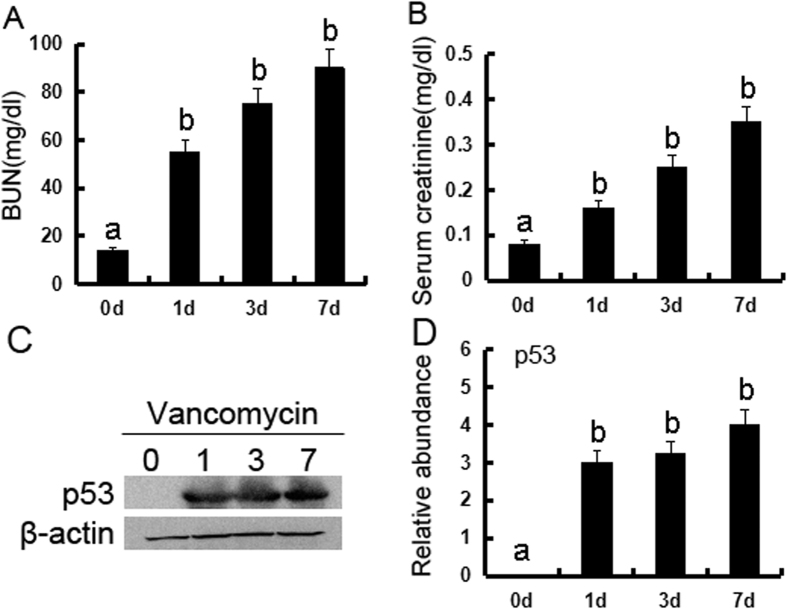
p53 is induced in VAN nephrotoxic AKI in mice. Male C57BL/6 mice were (**A–D**) injected with 400 mg/kg VAN (n = 8) for 0–7 days of examination. In **A** and **B**, blood samples were collected at the indicated time points to measure BUN and serum creatinine. In **C** and **D**, kidneys were harvested for immunoblot analysis of p53 and ß-actin (loading control). Data were expressed as means ± SD; the bars with different superscripts (a–c) in each panel were significantly different (*P* < *0.05*). Data are the representative of at least four separate experiments.

We first investigated whether p53 is induced during VAN nephrotoxic AKI. p53 was induced gradually in kidneys from day 1 to day 7, and accompanied by an increase in BUN and serum creatinine (Fig. 1A–D). These data for the first time indicate the induction of p53 in VAN nephrotoxic AKI.

### Deletion of p53 ameliorated renal dysfunction, renal injury, apoptosis, inflammation, cell cycle arrest, and cell death in VAN nephrotoxic AKI mice

**Figure 2:**
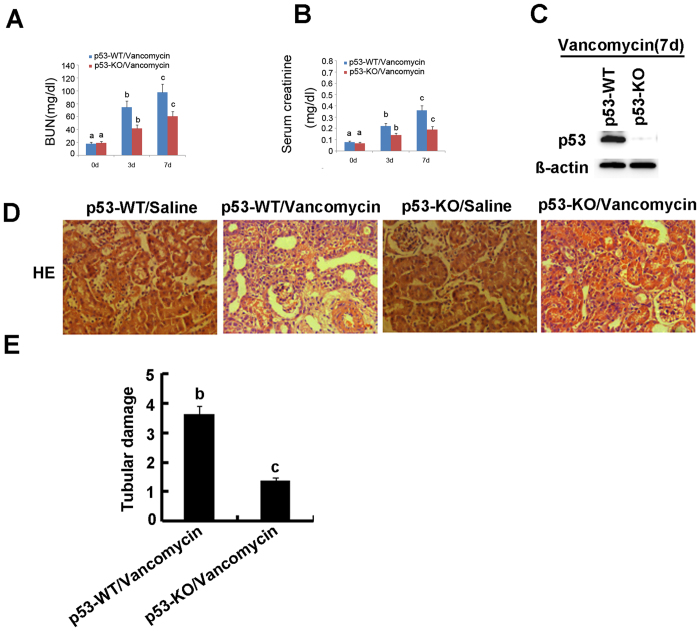
VAN induced AKI is attenuated in p53-KO mice. Wild-type and p53-KO littermate mice were injected with 400 mg/kg VAN (n = 8) or saline as control for 7 days. (**A** and **B**) Blood samples were collected for measurements of BUN and serum creatinine levels. (**C**) Kidneys were collected for immunoblot analysis of p53 and ß-actin. (**D**) Immunohistochemical staining of p53, Bar: 100 μM. (**E**) Tubular damage in VAN treated cortical tissues was semiquantified as pathologic scores. Data were expressed as means ± SD; the bars with different superscripts (a–c) in each panel were significantly different (*P* < *0.05*). Data are representative of at least four separate experiments.

To assess the role of p53 in the pathogenesis of VAN nephrotoxic AKI, the wild-type and p53-KO littermate mice were treated with or without VAN. In the non-VAN treatment group, levels of BUN and serum creatinine were similarly low. At day 3 and 7 of the VAN treatment, wild-type mice developed moderate renal failure, which was significantly reduced inp53-KO mice (Fig. 2A and B). By immunoblot analysis, p53 was completely abolished in p53-KO mice compared with WT mice after VAN treatment (Fig. 2C). Histologic analysis confirmed the VAN induced kidney tissue damage as in p53-WT mice, which was significantly ameliorated in p53-KO mice (Fig. 2D). In wild-type mice, the tubular damage score was 3.5 after VAN AKI, whereas the score was markedly decreased to 1.2 after VAN AKI for p53-KO tissues (Fig. 2E). Apoptosis plays an important role in the pathogenesis of AKI^20^, and p53 promotes apoptosis under cell stress^21^. The active caspase 3 and terminal deoxynucleotidyl transferase mediated digoxigenin deoxyuridine nick-end labeling (TUNEL) was used for assay of apoptosis in kidney cortical tissues. In the kidney tissues of saline-injected mice, positive cells of active caspase 3 and TUNEL was not detected. However, after VAN treatment, both of them were significantly induced in kidney cortical tissues in wild-type mice, but was markedly reduced in p53-KO mice (Supplementary Figure 1A). Furthermore, after VAN treatment, about 27% active caspase 3 was found in co-localization with TUNEL nuclei, and the number of co-localization of active caspase 3 and TUNEL in wild-type mice was significantly higher than the number of that in p53-KO mice. This observation was further verified by the number of apoptotic cells in cortical and outer medulla regions from independent experiments (Supplementary Figure 1B and C). We further examined the infiltration of inflammatory cells, the cell cycle arrest, and cell death in VAN nephrotoxic AKI. As shown in Supplemental Figures 2 and 3, VAN induced the infiltration of neutrophils and macrophages into wild-type kidney tissues, cell cycle arrest, and cell death, while they were reduced in p53-KO tissues.

### VAN induced p53 relative genes were suppressed in p53-KO mice

**Figure 3:**
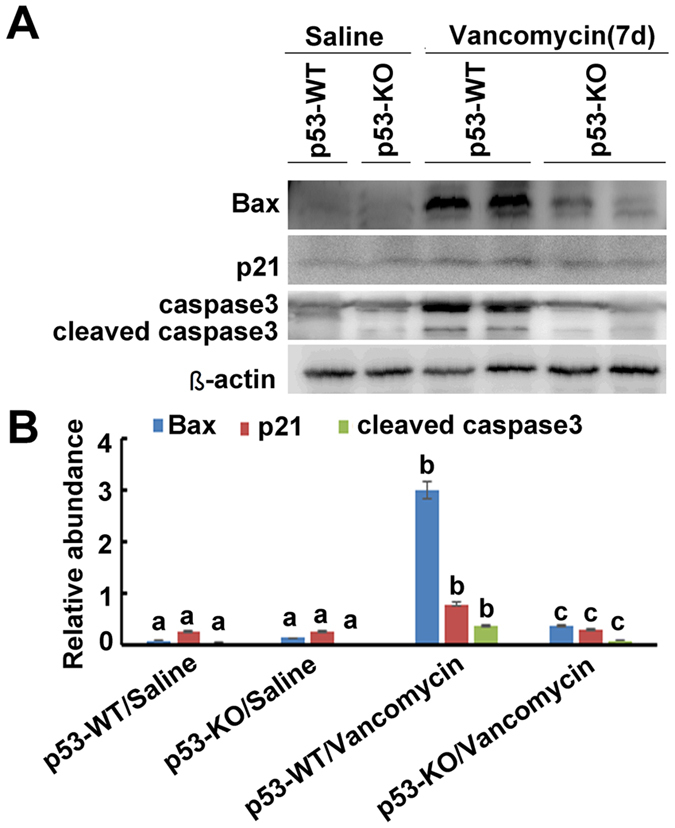
Induction of p53 target genes in VAN induced AKI is blocked in p53 KO mice. Wild-type and p53-KO littermate mice were injected with 400 mg/kg vancomycin (n = 8) or saline as control for 7 days. Whole tissue lysate was analyzed for p21, Bax, cleaved caspase 3, caspase 3, and ß-actin by using specific antibodies. (**A**) Representative immunoblot analysis. (**B**) Immunoblot signals were quantified by densitometry, and normalized with ß-actin. Data were expressed as means ± SD; the bars with different superscripts (a–c) in each panel were significantly different (*P* < *0.05*). Data are representative of at least four separate experiments.

Previous studies reported that several apoptotic genes induced by p53, such as p21 and Bax, were upregulated in cisplatin and ischemic induced AKI^11^,^22^. However, the expression of them in VAN induced AKI remains unclear. Therefore, we analyzed the expression of these genes to determine their dependence on p53. As shown in Fig. 3A, after VAN treatment, tissues of p53-KO mice showed less cleaved/active caspase 3, lower Bax and p21 expression, which were further substantiated by densitometric analysis of the immunoblots (Fig. 3B). Together, these data support that key cell death regulatory genes were induced by p53 during AKI.

### VAN induced miR-192-5p was blocked in p53-KO mice

**Figure 4:**
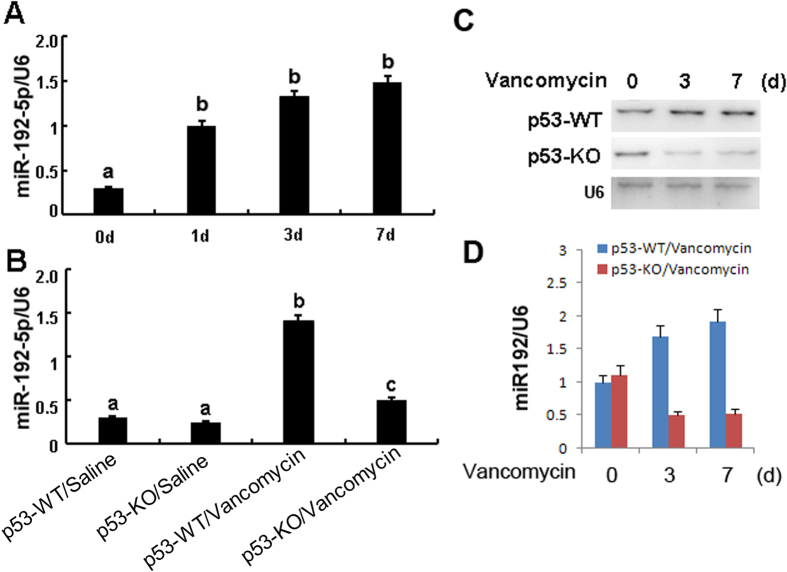
MiR192-5p expression is suppressed in p53 KO mice. Wild-type and p53-KO littermate mice were injected with 400 mg/kg VAN (n = 8) or saline as control for 0–7 days. Whole tissue lysate was analyzed for miR192–5p and U6 by Real-time PCR or Northern blot. (**A**) Real-time PCR showed the time-course of miR-192-5p expression levels in VAN induced AKI. (**B**) Real-time PCR showed miR192-5p expression is reduced in p53 KO mice. (**C**) Representative Northern blot analysis. (**D**) Northern blot signals were quantified by densitometry, and normalized with U6. Data were expressed as means ± SD; the bars with different superscripts (a–c) in each panel were significantly different (*P* < *0.05*). Data are the representative of at least four separate experiments.

Recent studies demonstrated that miR-192-5p mediated apoptosis in a few of tumor cell line^23^. To further reveal the in depth molecular mechanism of p53 for regulation of apoptosis, we focus on the miR-192-5p. Firstly, Real time PCR demonstrated that it was significantly increased on day 1 after VAN treatment, and gradually increased on day 3 and 7 (Fig. 4A). As shown in the non-VAN treatment group (Fig. 4B), level of miR-192-5p was similarly low in these mice, after VAN treatment, tissues of p53-KO mice showed low miR-192-5p compared to p53-WT mice. MiR-192-5p expression was further confirmed by Northern blot analyses, at day 3 and 7 after VAN treatment, it was markedly downregulated in p53-KO mice than in p53-WT mice (Fig. 4C and D).

### VAN induced p53 relative genes were suppressed by pifithrin-α in HK-2 cells

**Figure 5:**
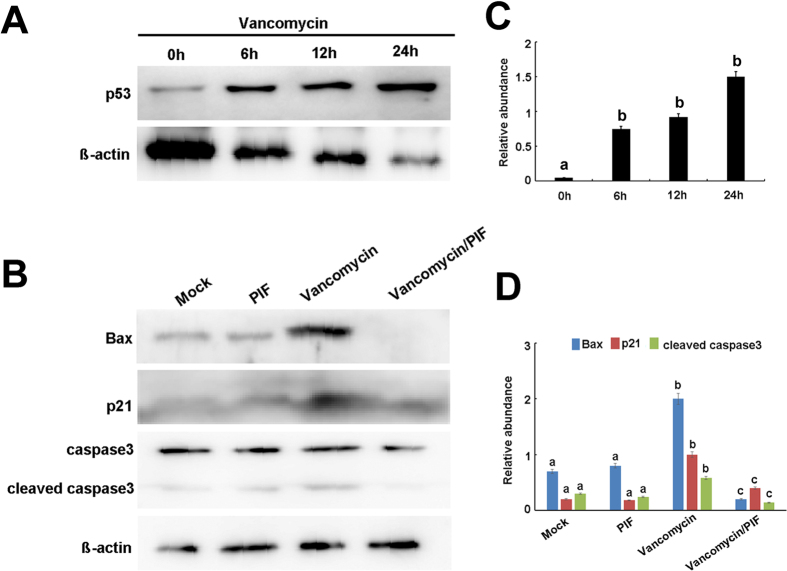
Pifithrin-α suppresses VAN induced p53 target genes in HK-2 cells. (**A**) HK-2 cells were treated with 4 mm/L VAN in presence or absence of 20 μM pifithrin-α for 24 h to collect lysate for immunoblot analysis of expression of p21, Bax, cleaved caspase 3, caspase 3, and ß-actin. (**B**) Immunoblot signals were quantified by densitometry, and normalized to internal actin control. Data were expressed as means ± SD; the bars with different superscripts (a–c) in each panel were significantly different (*P* < *0.05*). Data are representative of at least four separate experiments.

To further confirm the role of p53 in VAN induced AKI, HK-2 cells were used in this study. We first investigated whether p53 is induced after VAN treatment. p53 was induced in HK-2 cells gradually from 0 to 24 h, which were further confirmed by densitometric analysis of the immunoblots (Fig. 5A and C). As shown in Fig. 5B, after VAN treatment, pifithrin-α treated group showed less cleaved/active caspase 3, lower Bax and p21 expression, which were further demonstrated by densitometric analysis (Fig. 5D). Together, these data further suggest that p53 plays a critical role in VAN induced cell death.

### p53 was activated to promote apoptosis during VAN treatment

**Figure 6:**
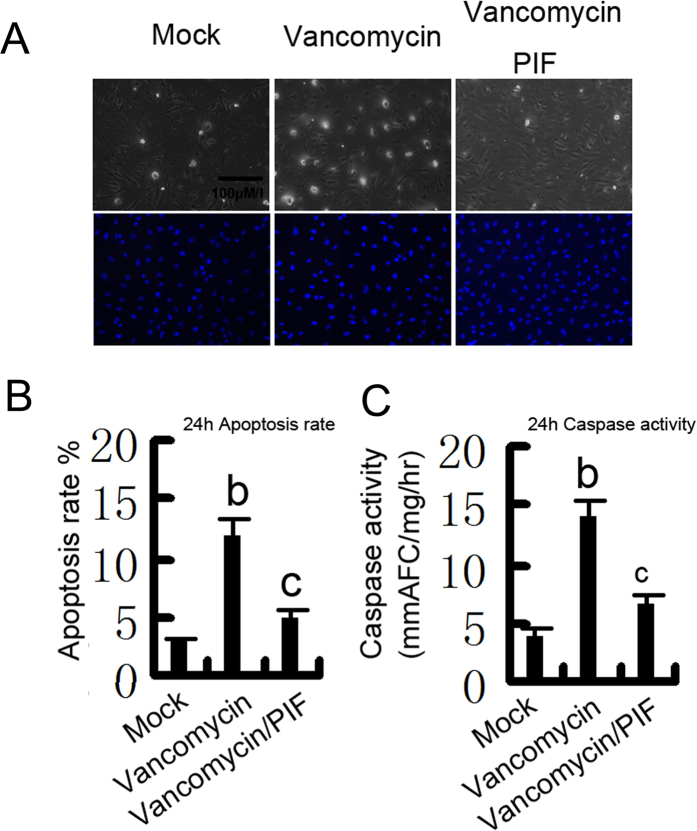
Effects of p53 inhibition on VAN induced apoptosis in HK-2 cells. HK-2 cells were treated with 4 mm/L vancomycin in presence or absence of 20 μM pifithrin-α for 24 h. (**A**) Morphology. Cells were stained with Hoechst33342. Cellular and nuclear morphology was recorded by phase-contrast and fluorescence microscopy. Bar: 100 μM/I. (**B**) Apoptosis was estimated as a percentage by counting the cells with typical apoptotic morphology. (**C**) Caspase activity. Cell lysate was collected for enzymatic assay of caspase activity. Data were expressed as means ± SD; the bars with different superscripts (a–c) in each panel were significantly different (*P* < *0.05*). Data are representative of at least four separate experiments.

We have shown that p53 has an important role in VAN induced AKI. To further confirm previous results, HK-2 cells were treated with pifithrin-α. As shown in Fig. 6A, vancomycin induced significant apoptosis in HK-2 cells, which was further inhibited by pifithrin-α. Analysis of apoptosis rate and caspase activity demonstrated similar result (Fig. 6B,C).

### p53 induced miR-192-5p for apoptosis during VAN treatment

**Figure 7:**
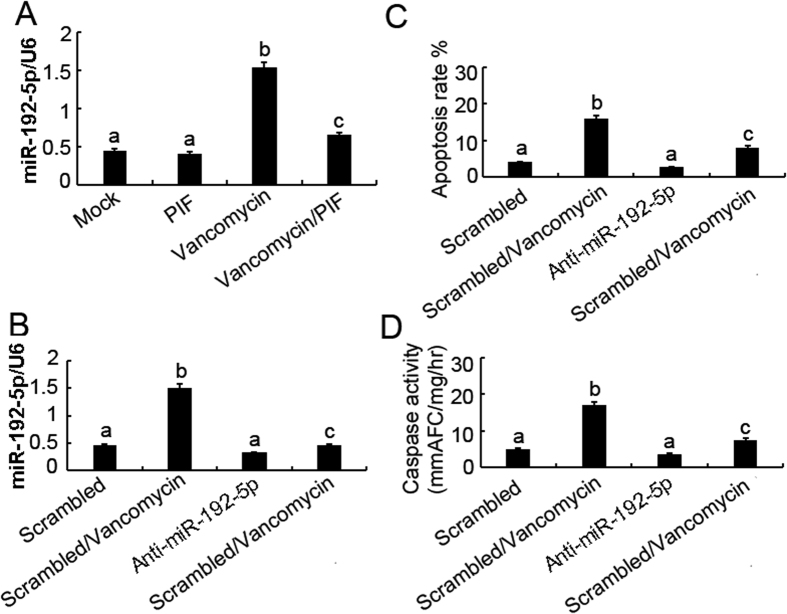
Blockade of miR-192-5p reduces VAN induced apoptosis in HK-2 cells. HK-2 cells were treated with 4 mm/L VAN in presence or absence of 20 μM pifithrin-α for 24 h, or transfected with 100 nmol/L LNA-modified antisense oligonucleotide of miR-192-5p (anti–miR-192-5p) or LNA-modified oligonucleotide of the scrambled sequence (scrambled). Cells were then left untreated or treated for 24 h with 4 mm/L vancomycin. (**A**) Effects of pifithrin-α on miR-192-5p induction during vancomycin treatment. Total RNA was isolated for real-time PCR analysis of miR-192-5p using specific primers. (**B**) Real-time PCR showed inhibition effects of anti–miR-192-5p on miR-192-5p expression. (**C**) Effects of anti–miR-192-5p on VAN induced apoptosis. Percentage of apoptotic cells was estimated by morphological methods. (**D**) Effects of anti–miR-192-5p on VAN induced caspase activation. Cell lysate was collected for an enzymatic assay of caspase activity. Data were expressed as means ± SD; the bars with different superscripts (a–c) in each panel were significantly different (*P* < *0.05*). Data are representative of at least four separate experiments.

To further confirm whether p53 induce the expression of miR-192-5p, pifithrin-α was used in current study. Real time PCR showed that VAN significantly induced miR-192-5p expression that was suppressed by pifithrin-α treatment (Fig. 7A). After VAN treatment, level of miR-192-5p was blocked by anti-miR-192-5p compared with the scramble control (Fig. 7B). As shown in Fig. 7C and D, VAN induced more apoptosis rate and caspase activity in HK-2 cells were inhibited by anti-miR-192-5p.

### Inhibition of miR-192-5p ameliorated renal dysfunction, renal injury in VAN nephrotoxic AKI mice

**Figure 8:**
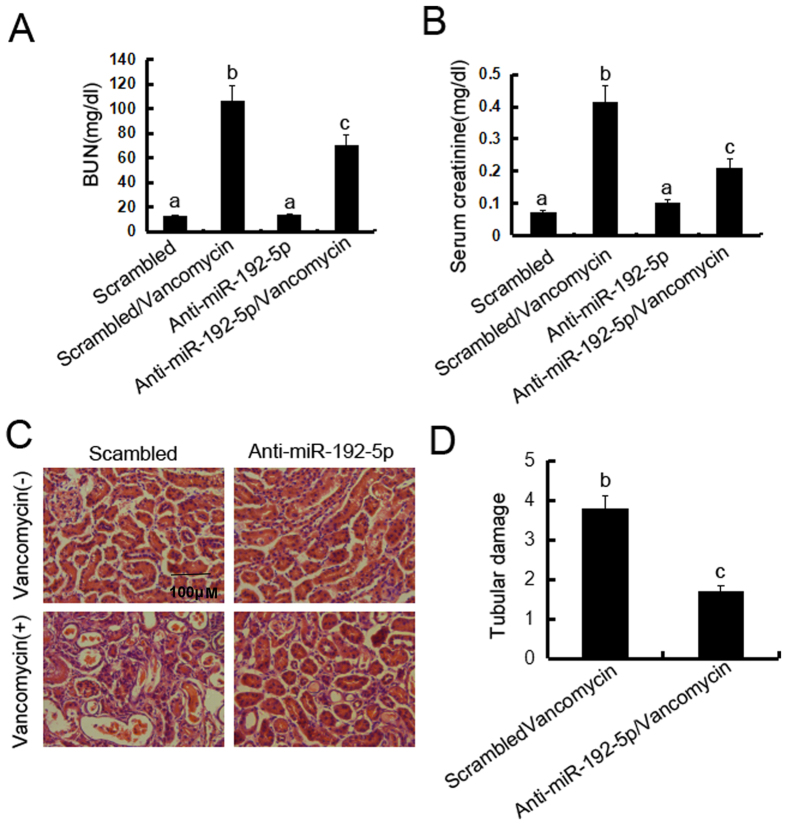
Blockade of miR-192-5p reduced VAN induced AKI. Male C57BL/6 mice were (**A–D**) injected with 400 mg/kg (n = 8) with or without 20 mg/kg LNA-modified antisense oligonucleotide of miR-192-5p (anti–miR-192-5p) or LNA-modified oligonucleotide of scrambled sequence (scrambled) for 7days of examination. In A and B, blood samples were collected to measure BUN and serum creatinine (**A** and **B**). (**C**) Kidney cortical tissues were stained with hematoxylin-eosin to show histology (original magnification, X200). (**D**) Tubular damage in VAN treated cortical tissues was semiquantified as pathologic scores. Data were expressed as mean ± SD; the bars with different superscripts (a–c) in each panel were significantly different (*P* < *0.05*). Data are representative of at least four separate experiments.

To assess the role of miR-192-5p in the pathogenesis of VAN nephrotoxic AKI, male C57BL/6 mice were injected with LNA-modified antisense oligonucleotide of miR-192-5p (anti–miR-192-5p) or LNA-modified oligonucleotide of the scrambled sequence (scrambled), levels of BUN and serum creatinine were similarly low in these mice, indicating normal renal function. At day 7 of VAN treatment, scrambled mice developed moderate renal failure, with 102.5 mg/dl BUN and 0.43 mg/dl serum creatinine, whereas anti–miR-192-5p mice had 65.9 mg/dl BUN and 0.22 mg/dl serum creatinine (Fig. 8A and B). Histologic analysis confirmed the VAN induced kidney tissue damage, which was significantly ameliorated in anti–miR-192-5p mice (Fig. 8C). In scrambled mice, the tubular damage score was 3.7 after vancomycin AKI, whereas the score was markedly decreased to 1.5 after vancomycin AKI for anti–miR-192-5p tissues (Fig. 8D). We further examined the cell cycle arrest in VAN nephrotoxic AKI. As shown in Supplemental Figure 4, VAN induced the cell cycle arrest, which was reduced in anti–miR-192-5p tissues.

## Discussion

A pathologic role of p53 in AKI is complex. Although inhibition of p53 by pifithrin-α or global p53-KO affords protection against cisplatin-induced mice AKI^11^,^12^,^14^,^15^, p53 inhibitors or global p53-KO mice enhanced ischemic induced mice AKI^19^. Our study has now shown that global p53-KO ameliorated VAN induced AKI. Hence, these studies indicate that p53 in different types of AKI models plays distinguished roles. Moreover, we have identified that miR-192-5p was regulated through p53 *in vivo and vitro*, and further revealed that p53 induced renal cell apoptosis via directly up regulation of miR-192-5p.

In present study, BUN levels appear to be high but a corresponding increase in creatinine. The degree of glomerular filtration function was reflected by Scr and BUN, latter is easily affected by extrarenal factors, such as high protein diet, dehydration, and high metabolism^24^,^25^, however, which is not sufficient to explain the inconsistence between BUN and creatinine in VAN induced AKI, a more reasonable explanation will need to be further studied in the future. Genotoxic stresses including oncogene activation, hypoxia and reactive oxygen species in both cancer and normal cells, induced DNA damage, and then activated p53 expression. p53 plays a critical role in cell cycle arrest, apoptosis and/or cell death^26^. Our result demonstrated that p53 was mainly induced in VAN induced AKI. As we know, VAN directly triggers mitochondrial apoptosis signaling in renal cell^4^. In this study, the renal cell apoptosis, inflammation, cell cycle arrest, and cell death induced by VAN were significantly reduced in global p53-KO mice and HK-2 cells treated with pifithrin-α, which was supported by our previous reports that p53 was involved in renal cell apoptosis, inflammation, cell cycle arrest and cell death in cisplatin and ischemic induced AKI^11^. However, Co-localization of active caspase3 and TUNEL is low. The end stage of severe irreversible cell damage was marked by TUNEL^27^. Caspase-3 is generally situated in the cytosol, during apoptosis, it is transported into the nucleus, and gains access to nuclear substrates^28^. Positive caspase-3 and negative TUNEL staining suggested that caspase-3 may be activated in cytosol, at very early stage during the initial steps of apoptosis, and before DNA fragmentation occur^29^. Our results for the first time demonstrated that inhibition of p53 ameliorated VAN induced AKI through reducing apoptosis, inflammation, cell cycle arrest, and cell death *in vivo and vitro*.

Previous studies showed the induction of several p53-regulated genes in cisplatin and ischemic-induced AKI^30^,^31^. Consistently, we confirmed the induction of Bax and p21 during VAN induced AKI. Importantly, the inductive response was largely suppressed in p53-KO mice, indicating that p53 was the key to the expression of these genes. Interestingly, although Bax are known to be proapoptotic, p21, a factor implicated in cell cycle, has a critical cytoprotective role in kidneys^32^, indicating that the gene induction by p53 was paradoxical in AKI. The possibility is that p21 induction by p53 activates defensive or cytoprotective mechanisms in initial stage^33^, however, sustained insult triggered pro-death gene expression^11^.

To further investigate the mechanisms how inhibition of p53 ameliorates VAN induced AKI, we focused on the miR-192-5p. Previous results have shown that miR-192 is involved in regulation of renal fibrosis^34^,^35^,^36^,^37^,^38^. To the date, the role of p53 in regulation of apoptosis remains controversial. Jin *et al*. reported that miR-192-5p provoked apoptosis by suppression of XIAP in tumor cell lines^23^,^39^. Although miR-192-5p might suppress apoptosis in A549 cells, inhibition of it did not alleviate apoptosis in the miR-192-5p non-responsive H1299 cells^23^. However, miR-192-5p suppresses apoptosis by targeting Bim in esophageal aquamous cell caicinoma^40^. In our study, we demonstrated that antagonizing VAN induced miR-192-5p by miRNA inhibitors decreased apoptosis in HK-2 cells. Furthermore, inhibition of miR-192-5p also suppressed VAN induced AKI, the cell cycle arrest was also reversed by miR-192-5p inhibitors, which was supported by the finding that miR-192 can enhance cell cycle arrest^41^. These data suggest that miR192-5p may act as an apoptosis promoter, and thus may be considered as a potential therapeutic target for VAN induced AKI. However, the role of miR-192-5p in apoptosis is dependent on the cell types/stress factors, miR192-5p as a potential new therapeutic target for other diseases could be further explored. Recent study reported that p53 could physically interact with the promoter region of miR-192-5p^42^. In current study, we also demonstrated that inhibition of p53 significantly suppressed miR-192-5p *in vitro and vivo*. To the date, we identified p53 promoted apoptosis by upregulation of miR-192-5p in HK-2 cells, revealing the involvement of this mechanism not only in HK-2 cells, but also in p53 knockout mice.

In conclusion, we demonstrated that p53 played a pivotal role in renal tubular injury, as evidenced by alleviation of VAN induced renal cell apoptosis using p53 inhibitor and global knockout p53 mice. In HK-2 cells and the mouse model, inhibition of p53 ameliorated VAN induced AKI through miR192-5p regulation. Our present study suggests the possibility that p53 may be a therapeutic target of AKI caused by VAN.

## Methods

### Reagents and Antibodies

Antibodies were purchased from the following sources: polyclonal anti-p53, Gr-1, F4/80, and active caspase3, and p-H3 from Cell Signaling Technology (Beverly, MA), polyclonal anti-p21, and anti-Bax (N-20) from Santa Cruz Biotechnology (Dallas, TX). All secondary antibodies were from Thermo Fisher Scientific. Carbobenzoxy-Asp-Glu-Val-Asp-7-amino-4-trifluoromethyl coumarin (DEVD- AFC), and 7-Amino-4-trifluoromethylcoumarin (AFC) were purchased from Enzyme Systems Products (Livermore, CA). Pifithrin-a was from EMD chemicals (Philadelphia, PA). PI staining kit and VAN was obtained from Sigma-Aldrich (St Louis, MO, USA). *In situ* the cell death detection kit was obtained from Roche Applied Science (Indianapolis, IN).

### Cell culture and treatments

HK-2 cells were cultured in Dulbecco’s modified Eagle’s medium (Sigma-Aldrich) supplemented with 10% fetal bovine serum, 0.5% penicillin, and streptomycin in 5% CO2 incubator at 37 °C. For Pifithrin-a treatment, HK-2 cells were treated with or without Pifithrin-a (20 μM) or vancomycin (4 mm/L) for 24 h. For transfection experiment, transfection of miR-192-5p analog (100 nM) or negative control (miR-neg; Sigma) were used.

### Animal model

p53 global knockout mice were purchased from Shanghai Biomodel Organism Science &Technology Development Co., Ltd (Shanghai, People’s Republic of China). The mice were intraperitoneally injected with a single dose of vancomycin at 400 mg/kg. In addition, the experiment on role of miR-192-5p, male C57BL/6 mice were injected by tail vein with or without 20 mg/kg LNA-modified antisense oligonucleotide of miR-192-5p (anti–miR-192-5p) or LNA-modified oligonucleotide of scrambled sequence (scrambled) for 7 days. The control group was administered with saline. Use of animal and the experimental protocols were in accordance with the guidelines and approved by the Institutional Committee for the Care and Use of Laboratory Animals of Second Xiangya Hospital, People’s Republic of China. Mice were sacrificed on day 7 after vancomycin or saline. The kidneys were harvested for various morphological and biochemical studies.

### Renal Function, Histology, and TUNEL Assay

BUN and serum creatinine were measured with commercial kits from Stanbio Laboratory^11^,^22^,^43^. For the histological analysis, kidney tissues fixed with 4% buffered paraformaldehyde were embedded in paraffin, and 4 μm thick sections were prepared. The sections were then stained with Hematoxylin-Eosin, followed by a blind examination. The score of tissue damage was assessed according to the percentage of damaged tubules: 0, no damage; 1, less than 25% damage; 2, 25–50% damage; 3, 50–75% damage; 4, more than 75% damage. The loss of brush border, tubular dilation, cast formation, and cell lysis, were the criteria of tubular damage. The *In Situ* Cell Death Detection Kit from Roche Applied Science was using for TUNEL assay. Cell death was stained by PI Kit^44^. For quantification, we randomly selected 10–20 fields from each tissue section to count the TUNEL-positive cells per millimeter^45^.

### Immunohistochemistry, immunofluorescence, and Immunoblot analysis

Immunohistochemical or immunofluorescence analyses were performed by using anti-p53, Gr-1, F4/80, or active caspase 3 according to the previous protocol^46^,^47^. Total numbers of positive cells for p53 (as identified by nuclear staining) was quantified by counting the number of stained cells per field. We collected 25–30 images of a kidney from each animal at X20 magnification. Immunoblot was carried out as previously described^11^,^22^,^48^. Briefly, cells or kidney tissues were treated with a lysis buffer (Sigma) containing phosphatase inhibitors (Calbiochem). Each well was loaded with equal amounts of proteins for electrophoresis using SDS–PAGE gel, followed by transferring to polyvinylidene fluoride membranes. Primary antibodies were incubated with membranes overnight at 4 °C, and probed by the horseradish peroxidase-conjugated secondary antibodies. Bands of target and internal control protein were separately outlined, and then grey level was analyzed using an image J software (NIH, USA), the grey ratio of target protein versus internal control protein was calculated.

### Real-Time PCR Analysis of miRNAs

Total RNA of kidney cortical tissues or HK-2 cells was extracted by the mirVanamiRNA isolation kit (Applied Biosystems/Ambion, Austin, TX) according to the manufacturer’s instruction. Using miRNAqRT-PCR Detection Kit (Ambion), forty nanograms of total RNA was reverse-transcribed to cDNA. Real-Time PCR was performed using the Taqman miRNA assay kit (Applied Biosystems) including the sequence-specific primers for cDNA synthesis and Taqman probes for real-time PCR. Quantification was done using ΔCt values.

### Northern Blot Analysis of miRNAs

The mirVanamiRNA isolation kit was used to extract total RNA. A denaturing 10% polyacrylamide gel was used to run ten micrograms of RNA, which was then transferred onto the Hybond-N + membrane (Amersham, Piscataway, NJ), subjected to UV light irradiation for 4 min and baked at 80 °C for 1 hour. Using ULTRA hyb-Oligo Hybridization Buffer (Applied Biosystems/Ambion), the membrane was pre-hybridized for 1 h, and subjected to hybridization with ^32^p-labeled antisense specific miRNA probe overnight at 37 °C. Then the membrane was washed in 2 × SSC buffer (0.1% SDS) and exposed to x-ray film at −80 °C.

### Statistical analysis

Data were expressed as Mean ± SEM. One-way ANOVA followed by the Tukey’s post-hoc test was used to compare multiple treatment groups. Two-way ANOVA was used to assess the statistical significance of the differences between multiple treatment groups at different time points. Statistical significance was set at P < 0.05.

## Additional Information

**How to cite this article**: Chen, J. *et al*. p53 activates miR-192-5p to mediate vancomycin induced AKI. *Sci. Rep.* **6**, 38868; doi: 10.1038/srep38868 (2016).

**Publisher's note:** Springer Nature remains neutral with regard to jurisdictional claims in published maps and institutional affiliations.

## Supplementary Material

Supplementary Information
