# Supplementary material for: p53 activates miR-192-5p to mediate vancomycin induced AKI
Source: Sci Rep. 2016 Dec 12;6:38868. doi: 10.1038/srep38868 (PMC5150818; doi:10.1038/srep38868)
Supplement: Supplementary Information [file srep38868-s1.pdf]

p53 activates miR-192-5p to mediate vancomycin induced AKI

Jinwen Chen, Juan Wang, Huiling Li, Shixuan Wang, Xudong Xiang , Dongshan Zhang

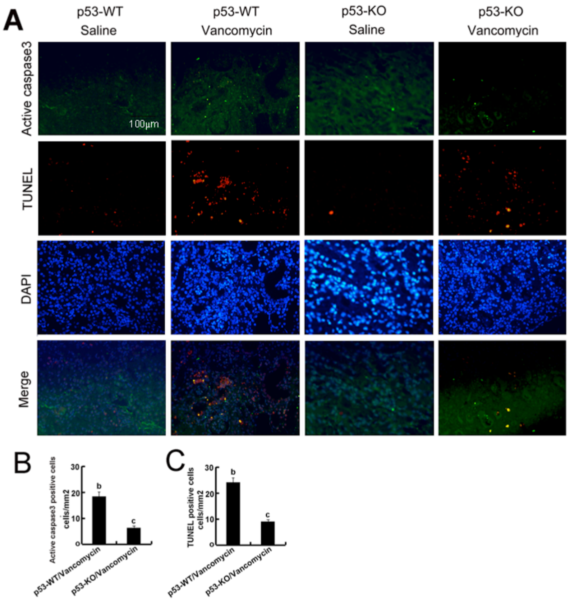

Supplementary Figure 1.Wild-type and p53-KO littermate mice were injected with 400 mg/kg VAN (n=8) or saline as control for 7 days. (A) Active caspase3 and TUNEL assay to reveal apoptosis (original magnification, ×200). Quantification of Active caspase3 (B) and TUNEL-positive cells (C) in VAN treated cortical tissues. Data were expressed as means ± SD; the bars with different superscripts (a-c) in each panel were significantly different ($P<0.05$). Data are representative of at least four separate experiments.

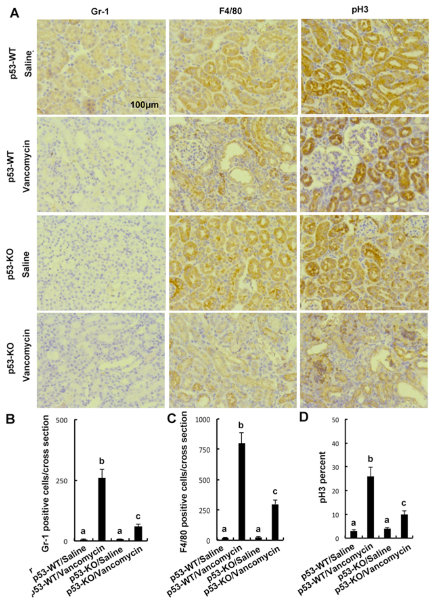

Supplementary Figure 2. Infiltration of neutrophils and F4/80s, and expression of p-H3 in VAN induced AKI in p53 KO and wild-type mice. Wild-type and p53-KO littermate mice were injected with 400 mg/kg vancomycin (n=8) or saline as control for 7 days. (A) Immunohistochemical staining of neutrophils, macrophages, and expression of p-H3 (original magnification ×200). (B) Quantitation of positive staining cells per cross-sectional area. Data were expressed as means ± SDs; the bars with different superscripts (a-c) in each panel were significantly different (P<0.05). Data are representative of at least four separate experiments.

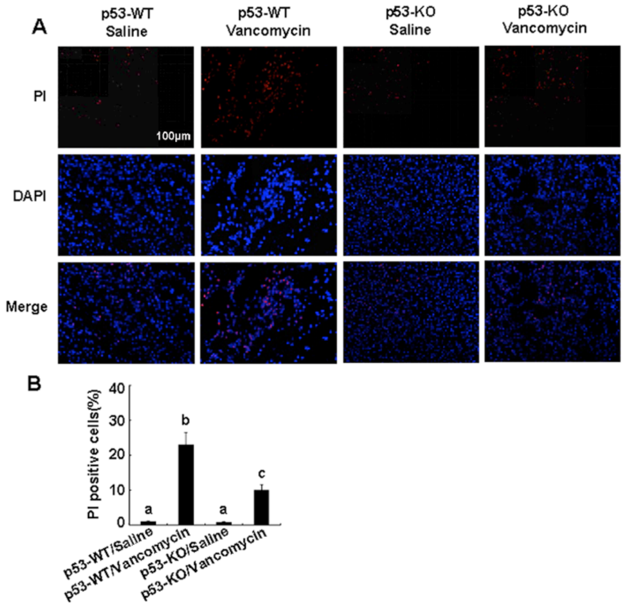

Supplementary Figure 3. Necrotic cell death was reduced in p53 KO mice. Wild-type and p53-KO littermate mice were injected with 400 mg/kg vancomycin (n=8) or saline as control for 7 days. (A) Representative images of kidney sections are shown, the Red, PI-positive tubular cell nuclei; blue, DAPI-stained nuclei (n=8). (B) Quantitative analysis of PI positive cells is shown. Data were expressed as means ± SDs; the bars with different superscripts (a-c) in each panel were significantly different ($P<0.05$). Data are representative of at least four separate experiments.

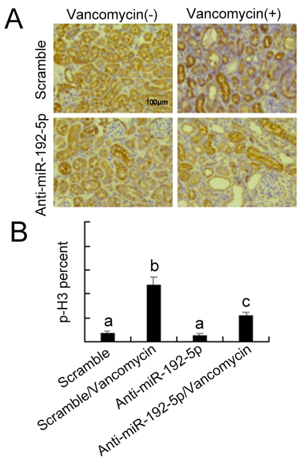

Supplementary Figure 4. Blockade of miR-192-5p reversed cell cycle arrest in VAN induced AKI. Expression of p-H3 in VAN induced AKI in p53 KO and wild-type mice. Male C57BL/6 mice were (A-F) injected with 400 mg/kg VAN (n=8) with or without 20mg/kg LNA-modified antisense oligonucleotide of miR-192-5p (anti–miR-192-5p) or LNA-modified oligonucleotide of scrambled sequence (scrambled) for 7 days of examination. (A) Immunohistochemical expression of p-H3 (original magnification X 200). (B) Quantitation of positive staining area per cross-sectional area. Data were expressed as means ± SDs; the bars with different superscripts (a-c) in each panel were significantly different ($P<0.05$). Data are representative of at least four separate experiments.
